# Development of a nested PCR assay for detecting *Colletotrichum siamense* and *Colletotrichum fructicola* on symptomless strawberry plants

**DOI:** 10.1371/journal.pone.0270687

**Published:** 2022-06-28

**Authors:** Pei-Che Chung, Hung-Yi Wu, Yi-Chia Chen, Ting-Hsuan Hung, Chia-Lin Chung

**Affiliations:** 1 Miaoli District Agricultural Research and Extension Station, Council of Agriculture, Executive Yuan, Miaoli County, Taiwan; 2 Department of Plant Pathology and Microbiology, National Taiwan University, Taipei City, Taiwan; University of Wisconsin Milwaukee, UNITED STATES

## Abstract

Anthracnose is a major disease of strawberry that seriously impacts the strawberry industry. To prevent the spread of anthracnose through symptomless plants, it is important to detect pathogenic *Colletotrichum* spp. at the latent infection stage in the nursery. Previous PCR-based methods developed for the diagnosis or detection of *Colletotrichum acutatum* and *Colletotrichum gloeosporioides* have used primers targeting the internal transcribed spacer region of ribosomal DNA, β-tubulin gene, or mating type gene. In this study, to specifically detect *Colletotrichum siamense* and *Colletotrichum fructicola*, the most predominant and virulent *Colletotrichum* species causing strawberry anthracnose in Taiwan, we conducted a comparative genomics analysis of 29 *Colletotrichum* spp. and identified a non-conserved 1157-bp intergenic region suitable for designing specific primers for a nested PCR assay. *In silico* analysis and actual tests suggested that the new nested PCR assay could detect pathogenic *C*. *siamense* and *C*. *fructicola*, but not other strawberry pathogens (*Botrytis* sp., *Fusarium* spp., *Neopestalotiopsis rosae*, and *Phytophthora* sp.) or ubiquitous saprophytes (*Fusarium* spp. and *Trichoderma* spp.). The inner to outer primer ratio was optimized to 1:10 to eliminate unexpected bands and enhance the signal. The assay could detect as little as 1 pg of *C*. *siamense* genomic DNA, which corresponds to ~15 cells. Application of the new detection assay on 747 leaf samples collected from 18 strawberry nurseries in 2019 and 2020 showed that an average of 20% of strawberry mother plants in Taiwan were latently infected by *C*. *siamense* or *C*. *fructicola*. The newly developed assay is being applied to facilitate the production of healthy strawberry runner plants in Taiwan.

## Introduction

Strawberry (*Fragaria *×* ananassa* Duch.) is an economically important small fruit crop that can grow in temperate, sub-tropical, and tropical regions around the world. The average strawberry cultivation area in Taiwan was about 514 ha from 2016 to 2020 [1], and Miaoli County is the predominant strawberry production region (~90% of the total cultivated area), where the major cultivars are ‘Taoyuan No. 1,’ and ‘Xiang-Shui’. Farmers in Taiwan usually propagate strawberry runner plants from mother plants in the spring and summer. The high temperature and intermittent heavy rains during the monsoon and typhoon seasons create environmental conditions conducive to infectious disease and epidemics. In recent years, strawberry anthracnose has become a more serious problem, causing the death of about 20%–40% of plants [2]. A high percentage of severely diseased plants are removed during the nursery stage or within 1–2 months after transplanting, which eventually leads to considerable economic loss.

*Colletotrichum* is a fungal genus containing important plant pathogens causing anthracnose diseases of various economically valuable crops [3]. Several *Colletotrichum* species can infect strawberry, causing leaf spot, crown rot, stolon spot, petiole spot, and fruit rot [4]. Based on traditional morphological characteristics, the major pathogens of strawberry anthracnose are considered to be *C*. *fragariae*, *C*. *gloeosporioides*, and *C*. *acutatum* [5,6]. Recent advances in molecular phylogenetics revealed that *C*. *gloeosporioides* and *C*. *acutatum* are highly diverse species complexes and can be classified into several species [7,8]. *C*. *nymphaeae* in the *C*. *acutatum* species complex was identified as the major pathogen of strawberry anthracnose in the UK and USA [9,10]; *C*. *fructicola* (*C*. *gloeosporioides* species complex) in Japan (Chiba Prefecture) and China (Zhejiang province) [11,12]; and *C*. *siamense* (*C*. *gloeosporioides* species complex) in China (Hubei province) [13]. In Taiwan, a recent investigation of the diversity of infectious agents causing strawberry anthracnose in Miaoli, Hsinchu, Nantou, and Chiayi Counties from 2010 to 2018 revealed five *Colletotrichum* spp., i.e., *C*. *siamense* (75% of all isolates), *C*. *fructicola* (11%), *C*. *karstii* (6%), *C*. *miaoliense* (6%), and *C*. *boninense* (2%) [14].

*Colletotrichum* is a hemibiotrophic pathogen that can remain in a latent state in the host tissues. Its conidium germinates to form an appressorium on the plant surface, which is followed by penetration peg invasion of the epidermal cells and the production of primary hyphae (biotrophic phase). The time required from the adherence of the conidia to successful infection can be within 48 hours [15,16]. At this stage, no visible difference can be observed between healthy and infected tissues [17,18]. However, *Colletotrichum* is capable of producing secondary conidia [19,20], which makes the latently infected but asymptomatic mother plants an important inoculum source for runner plants in the nursery. Latently infected runner plants can also be an inoculum source in production fields [21–23]. After the lifestyle transition from biotrophy to necrotrophy, this polycyclic pathogen produces masses of conidia, spreads rapidly by rain splash, and causes epidemics under suitable conditions in the field [22].

The use of pathogen-free strawberry mother plants and their propagules is a critical step for disease control in the nursery and production fields and thus the reduction of losses caused by anthracnose [22]. Several methods for diagnosing latent infection or distinguishing *Colletotrichum* spp. infecting strawberry have been developed [21,23–33]. For instance, culture-based methods involve incubation of strawberry tissues after surface sterilization with ethanol or killing with paraquat or freezing [24,29–31]. These treatments promote the necrotrophic growth of the latently infected pathogens, resulting in the formation of yellow to orange conidiomata after 1–2 weeks of incubation. Culture-based methods are simple and convenient but a long time is required for sporulation; in addition, a great deal of experience is required to correctly differentiate *Colletotrichum* spp. from other fast-growing fungi with similar morphology [29,30]. Faster and more accurate PCR-based detection methods have also been developed, using specific primers targeting the internal transcribed spacer (ITS) region of ribosomal DNA [25,27,28,32], β-tubulin (*TUB2*) gene [21], or mating type gene *MAT1-2* [33]. During latent infection with *Colletotrichum* spp., the number of pathogen cells at the biotrophic phase is very low [34,35], and only methods with high sensitivity (e.g., nested PCR or qPCR) can be applied to detect such a low amount of target DNA.

**Fig 1:**
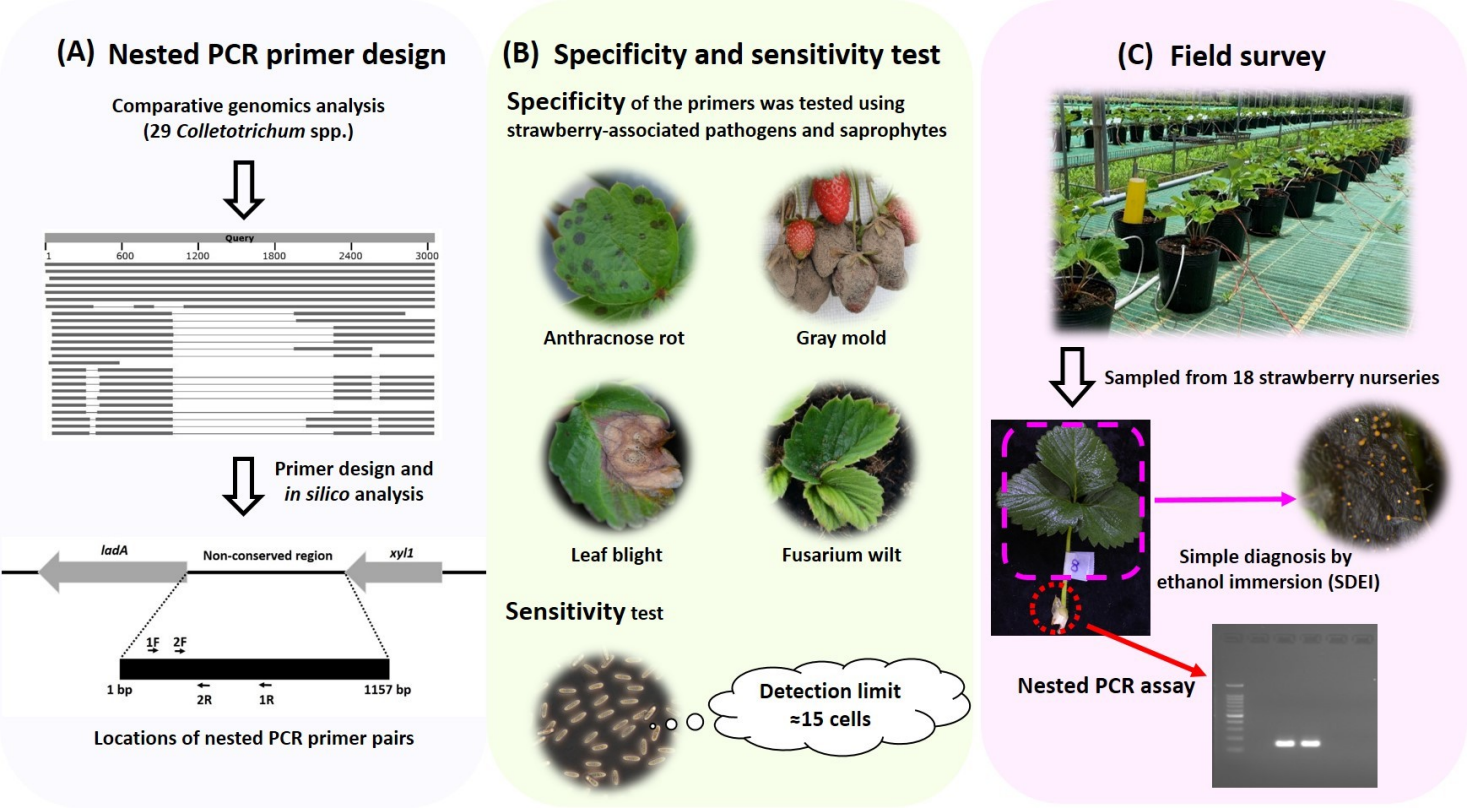
Workflow diagram for the nested PCR primer design, specificity and sensitivity tests, and field survey in this study. (A) Comparative genomics analysis and *in silico* analysis were conducted to identify non-conserved regions suitable for designing nested PCR primers. (B) Specificity and sensitivity tests of nested primers. Specificity was determined by testing the ability of primers to amplify strawberry-associated pathogens and saprophytes. The detection limit was ~15 cells of *C*. *siamense*. (C) Samples collected from 747 mother plants in 18 strawberry nurseries were assayed by the nested PCR and simple diagnosis by ethanol immersion (SDEI) methods.

PCR-based detection methods were previously designed to detect the anthracnose pathogens *C*. *acutatum* and *C*. *gloeosporioides* in strawberry [21,23,25,28,32,33,36]. However, *C*. *acutatum* and *C*. *gloeosporioides* are now considered a species complex based on recent evidence from multilocus molecular phylogenetic analyses [7,8], so the previous methods may not be able to distinguish current taxonomic species. This study aimed to develop a highly sensitive and specific method applicable for detecting the anthracnose pathogens on symptomless strawberry plants in Taiwan (the workflow of this study is shown in Fig 1). Due to the short period of time required for pathogenic *Colletotrichum* spp. to invade host tissues [15,16], the assay mainly targets the pathogens in the latent infection stage, although a small number of pathogens present on the host surface cannot be excluded. *C*. *siamense* and *C*. *fructicola*, the most predominant and virulent *Colletotrichum* species causing strawberry anthracnose in Taiwan [14], were targeted. Comparative genomics analysis of 29 available *Colletotrichum* spp. was conducted to search for a non-conserved region suitable for designing primers for a nested PCR assay (Fig 1A). *In silico* analysis and specificity tests were conducted to rule out detection of other pathogenic and saprophytic fungi frequently isolated from strawberries (Fig 1B), and the ratio of outer and inner primers used in the nested PCR were optimized to eliminate unexpected PCR products. To verify the new method and investigate the latent infection of strawberry plants by *Colletotrichum* spp. in Taiwan, a field survey was conducted on 747 asymptomatic mother plants in 18 strawberry nurseries (Fig 1C). As the production of strawberry runner plants is moving from propagation by small farmers toward professional propagation, it is expected that the highly specific and sensitive new method developed here will help reduce the disease incidence in mother plants, thereby increasing the rate of healthy runner plants in Taiwan.

## Materials and methods

### Fungal isolation and cultivation

The isolates of *Botrytis* sp. and *Colletotrichum* spp. [14], *Fusarium* spp., *Phytophthora* sp., and *Neopestalotiopsis rosae* [37], and *Trichoderma* spp. [38] used in this study are listed in S1 Table. In addition to five species causing strawberry anthracnose (*C*. *siamense*, *C*. *fructicola*, *C*. *karstii*, *C*. *miaoliense*, and *C*. *boninense*) [14] and *N*. *rosae*, which causes strawberry leaf blight and crown rot [37], we isolated *Botrytis* sp. from strawberry fruit showing gray mold, *Fusarium* spp. from the root, crown, and nearby soil of diseased plants showing typical Fusarium wilt symptoms, and *Phytophthora* sp. from the root of a wilted strawberry plant. A *Fusarium* sp. and *Trichoderma* sp. isolated from the symptomless runner and petiole of a strawberry plant and a *Trichoderma asperellum* isolate collected from the rhizosphere soil of grape were also included. Fungal isolation from host tissues was conducted as described by Chung et al. (2020) [14]. Tissues approximately 3 x 3 mm in area were surface sterilized with 0.5%–1% sodium hypochlorite, rinsed with sterile deionized water three times, then placed onto 1.5% water agar at 25°C. *Fusarium* isolation from soil was carried out by mixing 10 g of soil with 90 ml of 0.05% agar solution, then evenly spreading 200 μl of 10-fold serial dilutions on FoG1 medium (*Fusarium* colonies are purple on FoG1 medium) [39]. After 2 to 3 days of incubation, extended single hyphal tips from tissues were transferred to potato dextrose agar (PDA, BD Difco) and incubated for 5–7 days at 25°C under a 12-h/12-h light/dark photoperiod. Fungal isolates were identified to the genus level by morphological characteristics and ITS sequences (as described below).

### DNA extraction and sequence alignment

Genomic DNA was extracted from strawberry leaves or petioles using a plant genomic DNA extraction mini-prep system (VIOGENE) according to the procedures provided by the manufacturer. For extraction of fungal genomic DNA, the mycelium collected from a 7-day-old colony grown on PDA was frozen in liquid nitrogen and ground to a fine powder with a sterile mortar and pestle. The ITS was amplified with ITS1/ITS4 primers [40]. Amplicons were bidirectionally sequenced on an ABI 3730 DNA analyzer (Tri-I Biotech, Taiwan), and the sequences were used as queries in blast searches against the NCBI GenBank nr/nt database (blast.ncbi.nlm.nih.gov).

### Target region selection and primer design

To identify ideal regions for primer design, we searched for non-conserved regions located in between two conserved regions in the genomes of *Colletotrichm* spp. The conserved regions were used to design primers to sequence the internal non-conserved regions, which are highly diverse and can be used to distinguish *Colletotrichum* species. Based on the initial results of blastn searches against 29 *Colletotrichum* genome sequences (S2 Table) using the ITS, chitin synthase (*CHS-1*), actin (*ACT*), *TUB2*, calmodulin (*CAL*), and intergenic region between the *Apn2* DNA lyase and *MAT1-2* (ApMAT) genes (sequences obtained from our previous study [2]) as queries, *C*. *gloeosporioides* strain 30206, *C*. *gloeosporioides* Cg-14, and *C*. *fructicola* Nara gc5 (which was designated *C*. *gloeosporioides* before 2018) were the closest strains to *C*. *siamense* ML133. Note that the primer design for this study was conducted in 2017, at a time when the genome sequence of *C*. *siamense* was not available (*C*. *siamense* ICMP18578 was released in 2019). Therefore, *C*. *gloeosporioides* 30206 was used as a query to search against the genomes of 26 *Colletotrichum* species (all strains in S2 Table except *C*. *gloeosporioides* Cg-14, *C*. *gloeosporioides* 30206, and *C*. *fructicola* Nara gc5). All genome sequences were downloaded from the NCBI genome database [https://www.ncbi.nlm.nih.gov/genome/], and the genome blast was performed using BLAST Command Line Applications [41] following the user manual [https://www.ncbi.nlm.nih.gov/books/NBK279690/]. The blastn parameters were set to word size 28, e value <10^−5^, and output format 5. The output file was parsed using Python [42]. The 500- to 2500-bp non-conserved regions between conserved hit regions (hereafter referred to as ‘spacers’) were selected. The 1000-bp upstream and 1000-bp downstream sequences of each selected spacer were blasted against the genome sequences of 29 *Colletotrichum* spp. (blastn word size 28, e value <10^−100^). Spacers of 1000–1500 bp in length were selected from among those with upstream and downstream sequence hit numbers ≥ 50. Candidate spacers were checked manually and a region suitable for designing high-quality primers for a nested PCR assay was selected. The identified spacer region in *C*. *siamense* ML133 was sequenced by the primer pair 5’-TTGGCCTGCGCTTCAACGAC-3’ (forward) and 5’-AACTCACCCGCAAACACCAGT-3’ (reverse). Primers for the first PCR (outer primers) and second PCR (inner primers) were designed based on the spacer sequences. Primers with high scores and that were compatible with each other were chosen using Oligo 7 software [43]. Furthermore, nested PCR primer candidates were blasted against *Fragariae* x *ananassa* (NCBI accession No. PRJDB1477) and other pathogen/microbial genomes, including *Fusarium* spp. (S3 Table), *Trichoderma* spp. (S4 Table), and strawberry pathogens *Botrytis cinerea*, *Phytophthora cactorum*, and *Xanthomonas fragariae*, to rule out possible non-target reactions *in silico*. The primers with lower hit numbers and lower e values were chosen.

### Specificity and sensitivity of the nested PCR assay

Fifteen fungal isolates including pathogens and saprophytes isolated from strawberry or soil (S1 Table) and three strawberry cultivars (i.e., ‘Taoyuan No. 1’, ‘Xiang-Shui,’ and ‘Miaoli No. 1’) were used for evaluation of the specificity of the nested PCR assay. The primers targeting the ITS (ITS1/ITS4 [40]) and *ACTIN* gene (Actin-F/Actin-R [44]) were used to test the quality of fungal and strawberry DNA, respectively. Each PCR reaction was performed in a 50-μl mixture containing 2.5 U Taq polymerase (Prime Taq, GenetBio). For ITS and *ACTIN*, each reaction contained 1–20 ng DNA and 0.2 μM of each primer. For the nested PCR assay, the first PCR reaction contained 1–20 ng DNA and 0.02 μM of each outer primer (Col_nest-1F/Col_nest-1R), and the second PCR reaction contained 1 μl of the first PCR product and 0.2 μM of each inner primer (Col_nest-2F/Col_nest-2R) (primer sequences in Table 1). Different ratios of outer primers to inner primers (1:1, 1:2, 1:5, 1:10, 1:20, 1:50, and 1:100) were tested and 1:10 was found to be optimal. The conditions for the first PCR were an initial denaturation at 94°C for 5 min, followed by 40 cycles of 94°C denaturation for 30 sec, 55°C annealing for 30 sec, and 72°C extension for 30 sec with a final cycle of 72°C for 5 min. The conditions for the second PCR were an initial denaturation at 94°C for 1 min, followed by 30 cycles of 94°C denaturation for 30 sec, 55°C annealing for 30 sec, and 72°C extension for 30 sec with a final cycle of 72°C for 5 min. The PCR products were analyzed by electrophoresis on a 1.5% agarose gel in TAE buffer. Images were captured using a Fluorescent Gel Image System (FGIS-3, TopBio). The expected sizes of the first and second PCR products were 490 bp and 151 bp, respectively. To test the detection limit, genomic DNA of *C*. *siamense* was 10-fold serially diluted from 1 ng/μl to 10 fg/μl. The first and second PCR reactions were performed as described above.

**Table 1:** Nested PCR primers used in this study.

Name	Sequence	Product size (bp)	Note
Col_nest-1F	5’- ACAAACGGTGATCCTTTCGTC -3’	490	Outer primers for the first PCR
Col_nest-1R	5’- GGTGCCCCTCAACACGAAC -3’		
Col_nest-2F	5’- CTCCCAACCGGATAATCTGC -3	151	Inner primers for the second PCR
Col_nest-2R	5’- ACCGACCGGAACATAGATCACA -3’		

### Detection of *Colletotricum* spp. on symptomless strawberry plants in nurseries

From 2019 to 2020, 747 asymptomatic leaf samples (747 mother plants) collected from 18 strawberry nurseries in Hsinchu City, Miaoli County, Taichung City, and Nantou County were tested using the nested PCR and simple diagnosis by ethanol immersion (SDEI) methods [30] (Table 2). In these nurseries, the mother plants were reproduced from field plants by the farmers themselves. Previous studies showed that latent infection with *C*. *acutatum* and *C*. *gloeosporioides* was more frequently detected in the older leaves and petioles [22,29]. In this study, the oldest leaf was removed from the crown of each tested plant. An approximately 1-cm segment of the basal petiole was used for the nested PCR assay. The remaining leaf and petiole were used for the SDEI assay. The SDEI assay was conducted following the procedures in Ishikawa (2004) with modification [30]. In brief, the collected leaf samples were washed with tap water, rinsed with deionized water, and blotted dry on tissue paper. The abaxial and adaxial surfaces of the leaves were sprayed thoroughly with 75% ethanol. At 30–60 sec after spraying, the leaves were washed with deionized water once, rinsed with sterile water, and blotted dry on sterile tissue paper. The leaves were put into a plastic bag with a wetted cotton pad to maintain high humidity (> 90%). Leaves were incubated for 7–14 days at 28–30°C under a 12-h/12-h light/dark photoperiod.

**Table 2:** Field survey of *Colletotrichum* spp. on symptomless strawberry plants using the nested PCR and SDEI (simple diagnosis by ethanol immersion) assays.

Nursery site	Collection date	County/City	No. of leaf samples	Method	No. of positive samples	Detection rate (%)
1	2019/01/14	Miaoli County	50	Nested PCR	10	20
			50	SDEI	20	40
2	2019/03/08	Miaoli County	60	Nested PCR	9	15
			60	SDEI	16	27
3	2019/03/22	Hsinchu City	16	Nested PCR	2	13
			16	SDEI	5	31
4	2019/03/25	Taichung City	22	Nested PCR	6	27
			22	SDEI	14	64
5	2019/04/01	Miaoli County	50	Nested PCR	7	14
			50	SDEI	23	46
6	2019/04/09	Hsinchu City	6	Nested PCR	6	100
			6	SDEI	1	17
7	2019/05/01	Miaoli County	40	Nested PCR	1	3
			40	SDEI	2	5
8	2019/06/04	Miaoli County	41	Nested PCR	12	29
			41	SDEI	23	56
9	2019/06/21	Hsinchu City	7	Nested PCR	1	14
			7	SDEI	2	29
10	2019/07/04	Miaoli County	50	Nested PCR	19	38
			50	SDEI	11	22
11	2019/10/02	Nantou County	35	Nested PCR	1	3
			35	SDEI	6	17
12	2020/02/06	Miaoli County	50	Nested PCR	8	16
			50	SDEI	30	60
13	2020/02/13	Miaoli County	50	Nested PCR	2	4
			50	SDEI	21	42
14	2020/02/19	Miaoli County	50	Nested PCR	11	22
			50	SDEI	42	84
15	2020/03/04	Miaoli County	50	Nested PCR	0	0
			50	SDEI	14	28
16	2020/03/11	Miaoli County	50	Nested PCR	7	14
			50	SDEI	42	84
17	2020/04/08	Miaoli County	50	Nested PCR	11	22
			50	SDEI	45	90
18	2020/04/21	Miaoli County	70	Nested PCR	4	6
			70	SDEI	49	70
Total			747	Nested PCR	117	16
			747	SDEI	366	49
Average (per nursery)			41.5	Nested PCR	6.5	20
			41.5	SDEI	20.3	45

## Results

### Identification of a highly diverse intergenic region for primer design

Through comparative genomics analysis of 29 *Colletotrichum* spp. isolates, 19 non-conserved regions (1000–1500 bp in length) located between conserved regions were identified. After manually checking the sequences, a non-coding region was selected for primer design. This region in *C*. *siamense* ML133 was 1157 bp in length (sequences uploaded to GenBank under accession number ON350970), which has 95.77% and 94.65% identity to the corresponding regions in *C*. *gloeosporioides* 30206 and *C*. *siamense* Cg363, respectively. An alignment of the sequences of different *Colletotrichum* spp. is shown in S1 Fig. In the genome of *C*. *siamense* Cg363, the region is located between the L-arabinitol 4-dehydrogenase (*ladA*) and NAD(P)H-dependent D-xylose reductase (*xyl1*) genes. Using this region as template, 79 pairs of primers were designed. After performing blast searches against the sequences of *Fragariae* x *ananassa*, strawberry pathogens, and saprophytes, two primer pairs suitable for nested PCR were selected. The sizes of the first and second PCR products were 490 bp and 151 bp, respectively.

### Specificity and sensitivity of the nested PCR assay

**Fig 2:**
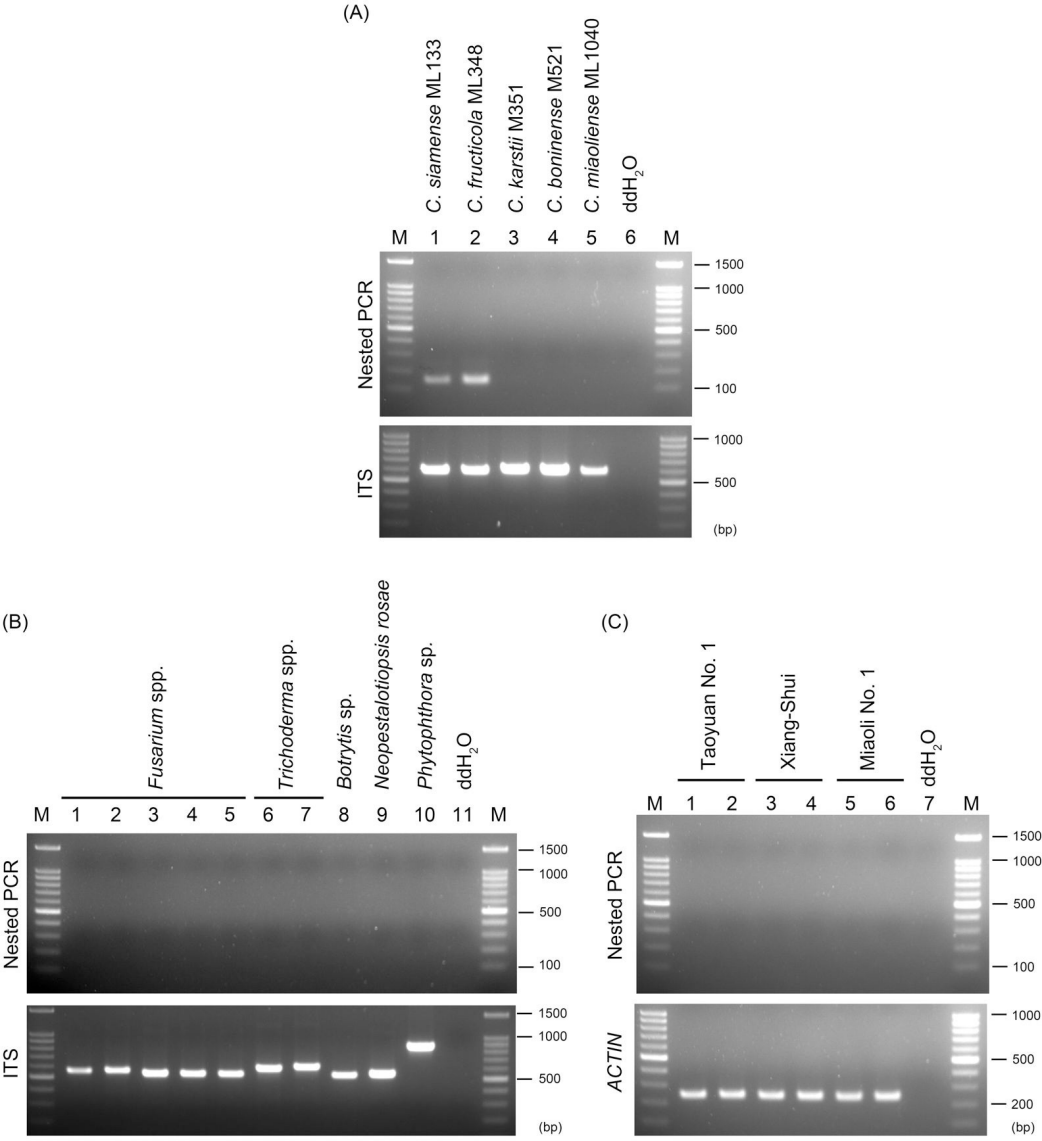
Specificity test of the nested PCR assay. (A) DNA of *Colletotrichum* spp. associated with strawberry anthracnose in Taiwan were used as template. *C*. *siamense* and *C*. *fructicola* are the most prevalent and virulent species, and *C*. *boninense*, *C*. *karstii*, and *C*. *miaoliense* are lowly pathogenic and present in a low percentage of strawberry plants. (B) DNA of pathogenic or saprophytic fungi isolated in the field were used as template. (C) DNA of different strawberry cultivars were used as template. The nested PCR assay was performed using primers Col_nest-1F/Col_nest-1R for the first PCR, and Col_nest-2F/Col_nest-2R for the second PCR. The quality of fungal and strawberry DNA was tested using primers targeting the ITS (ITS1/ITS4) and *ACTIN* (Actin-F/Actin-R), respectively. M, 100-bp DNA ladder (Faith Biotechnology Co., Ltd).

Five *Colletotrichum* spp. causing strawberry anthracnose in Taiwan and a selected set of microorganisms commonly isolated from strawberry or soil were used for the specificity test. Among the five pathogenic *Colletotrichum* spp., *C*. *siamense* ML133 and *C*. *fructicola* ML348 but not *C*. *karstii* ML351, *C*. *boninense* ML521, or *C*. *miaoliense* ML1040 were detectable (Fig 2A). The first and second PCR resulted in specific bands of the expected sizes (490 bp and 151 bp, respectively). The pathogenic fungi *Neopestalotiopsis rosae* ML2147, *Fusarium* spp., *Botrytis* sp., and *Phytophthora* sp. and saprophyte fungi *Fusarium* spp. and *Trichoderma* spp. were not detectable (Fig 2B). No signal was detected from three strawberry cultivars, ‘Taoyuan No. 1’, ‘Xiang-Shui’, and ‘Miaoli No. 1’ (Fig 2C). PCR products of the expected sizes were observed from the controls (fungal ITS and strawberry *ACTIN*) (Fig 2).

**Fig 3:**
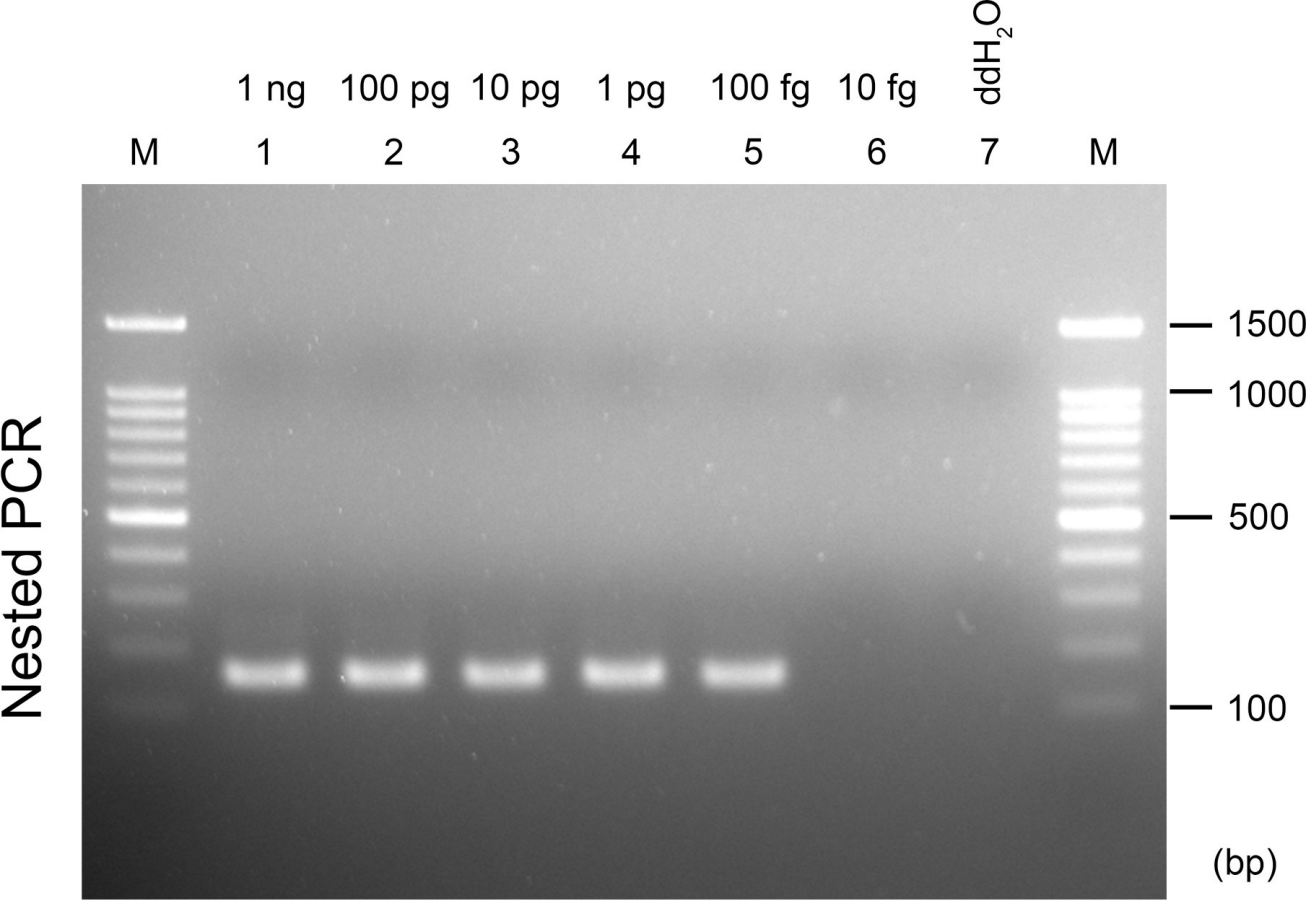
Sensitivity test of the nested PCR assay. Ten-fold dilutions (1 ng to 10 fg) of *C*. *siamense* ML133 genomic DNA were used as the template. The nested PCR assay was performed using primers Col_nest-1F/Col_nest-1R for the first PCR, and Col_nest-2F/Col_nest-2R for the second PCR. M, 100-bp ladder (Faith Biotechnology Co., Ltd).

In the sensitivity test, a bright and specific band was observed from the reactions using 1 ng, 100 pg, 10 pg, 1 pg, and 100 fg of the genomic DNA of *C*. *siamense* ML133. The product sometimes failed to be amplified when using 100 fg. The results showed that performing the first PCR with 40 cycles followed by the second PCR with 30 cycles can reliably detect as little as 1 pg genomic DNA (Fig 3), which corresponds to the DNA contents of ~15 cells of *C*. *siamense* (based on the genome size of *C*. *siamense* Cg363: ~62.9 Mb) [45].

### Optimization of the nested PCR assay by changing outer and inner primer ratio

**Fig 4:**
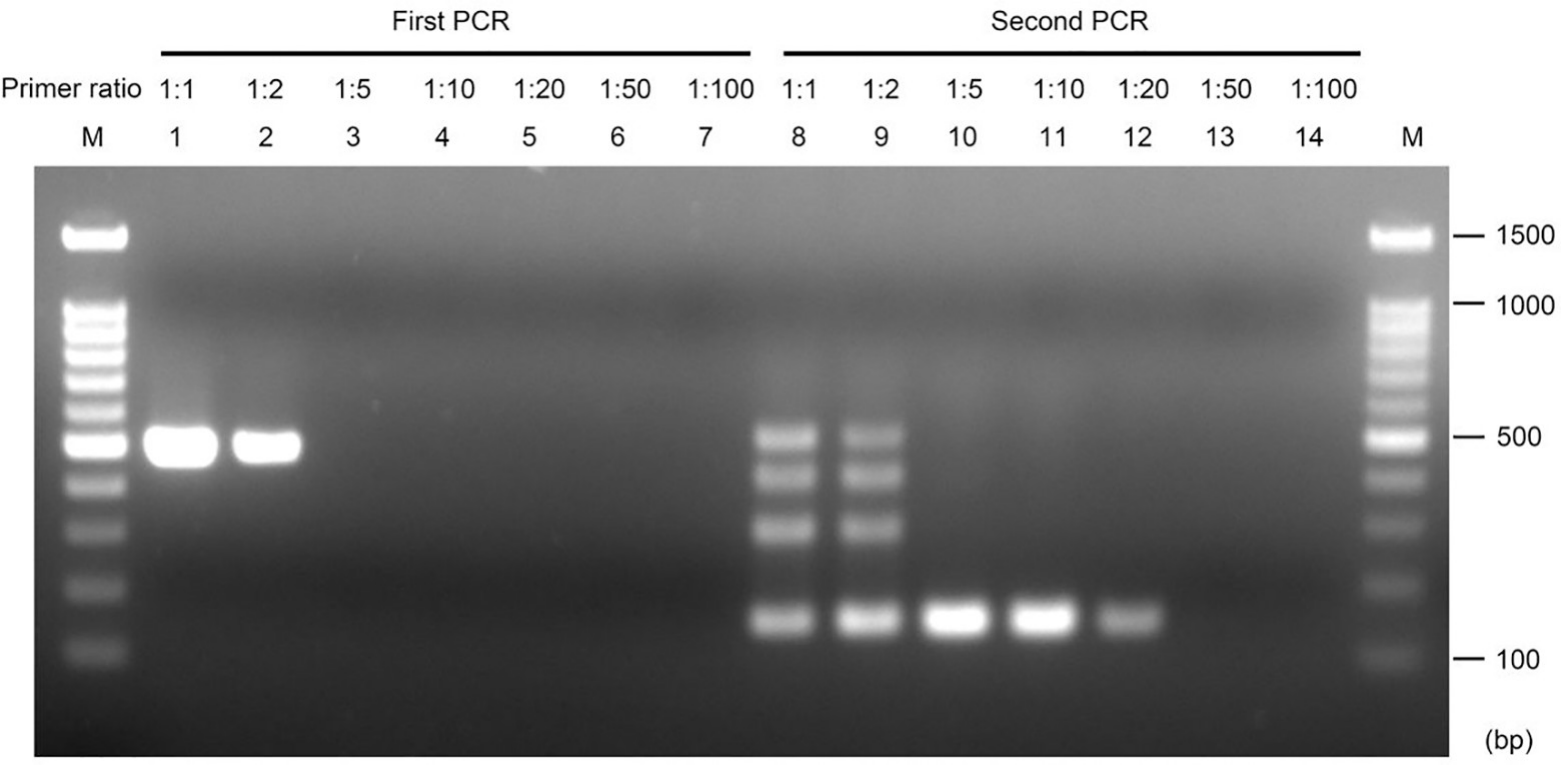
Optimization of the primer ratio for the nested PCR assay. Lanes 1–7, first PCR product; lanes 8–14, second PCR product. The nested PCR assay was performed using primers Col_nest-1F/Col_nest-1R for the first PCR, and Col_nest-2F/Col_nest-2R for the second PCR. Primer ratio represents the ratio of the concentrations of outer primers (first PCR) to inner primers (second PCR). The genomic DNA of *C*. *siamense* ML133 was used as the template. M, 100-bp ladder (Faith Biotechnology Co., Ltd).

It was observed that the nested PCR often resulted in four bands of approximately 500 bp, 400 bp, 250 bp, and 150 bp. The sizes of these bands suggested that they may have come from amplification directed by different combinations of the outer primers and inner primers. To reduce the non-target signals, we tested different ratios of the outer and inner primers ranging from 1:1 to 1:100. When the ratio was 1:1 or 1:2, all four bands appeared. A single band of the expected size (151 bp) was observed for ratios 1:5, 1:10, and 1:20 (Fig 4). Notably, the signal was stronger with ratios of 1:5 and 1:10.

### Field survey of *Colletotricum* spp. on symptomless strawberry plants in nurseries

From 2019 to 2020, 747 asymptomatic leaf samples collected from 18 strawberry nurseries were tested for strawberry anthracnose pathogens. The number of samples per nursery ranged from 6 to 70 (average 42 samples/nursery) (Table 2). Using our nested PCR assay, the detection rates ranged from 0% to 100% (average 20%); using the SDEI method [30], the detection rates ranged from 5% to 90% (average 45%) (Table 2). For the samples from 16 out of 18 nurseries, the detection rates from the nested PCR assay were lower than those from the SDEI method.

## Discussion

Symptomless runner plants carrying the inoculum of *Colletotrichum* spp. is an important route for the spread of strawberry anthracnose from the nursery to the field [23,33]. In Taiwan, *C*. *siamense* and *C*. *fructicola* are the most prevalent and virulent strawberry anthracnose pathogens [14]. Several PCR-based methods (conventional PCR, nested-PCR, and quantitative PCR) and culture-based methods (incubation of leaves treated with ethanol, herbicide, or freezing) have been developed for the diagnosis or detection of strawberry anthracnose [21,23–33]. However, a highly sensitive PCR-based detection method was previously not available for *C*. *siamense* and *C*. *fructicola*. In previous studies, PCR primers for detecting *Colletotrichum* spp. associated with strawberry were designed using the ITS, *TUB2*, or *MAT1-2* as the template [21,25,27,28,32,33]. However, among *Colletotrichum* spp., there is a high degree of sequence similarity between phylogenetic markers (ITS, *CHS-1*, *ACT*, *TUB2*, *CAL*, and ApMAT). The non-coding regions of *CHS-1*, *ACT*, *TUB2*, and *CAL* are more variable but too short (mostly < 100 bp) for designing highly specific nested PCR primers. In this study, we conducted comparative genomic analysis and identified an intergenic region between *ladA* and *xyl1* that was ideal for distinguishing *C*. *siamense* and *C*. *fructicola* from the other 16 *Colletotrichun* spp. *In silico* analysis and actual tests suggested that our newly developed nested PCR assay could detect pathogenic *C*. *siamense* and *C*. *fructicola*, but not other strawberry pathogens (*Botrytis* sp., *Fusarium* spp., *Neopestalotiopsis rosae*, and *Phytophthora* sp.) or ubiquitous saprophytes (*Fusarium* spp. and *Trichoderma* spp.). Although *C*. *boninense*, *C*. *karstii*, and *C*. *miaoliense* (the other three *Colletotrichun* spp. causing strawberry anthracnose in Taiwan) were not detectable, the assay is expected to detect most cases of latent infection that can lead to serious disease. *C*. *boninense*, *C*. *karstii*, and *C*. *miaoliense* are present in Taiwan at low percentage (total 14%) and cause tiny lesions (0.07–0.35 cm in diameter) only on wounded leaves even under a conducive high temperature (30°C) condition [14]. The assay can detect as low as 1 pg genomic DNA, which corresponds to ~15 cells of the pathogen. The ratio of the concentrations of nested primer pairs is critical for specificity [46,47], and the optimal outer and inner primer ratio for our nested PCR assay is 1:10. The high sensitivity and specificity of this assay allows the detection of trace amounts of pathogenic *C*. *siamense* and *C*. *fructicola*, without the problem of unexpected PCR products amplification.

Anthracnose spores are mainly disseminated by rain and overhead irrigation water. Older leaves at lower positions have more chances to be exposed to the pathogen inoculum; therefore, they are more likely to be infected than younger leaves at higher positions. In previous studies, old strawberry leaves/petioles were used as materials for detecting latent anthracnose infection [22,29]. Since older leaves are often removed by farmers for pest control purposes, they are good materials available all year round for detecting the source of pathogen inoculum.

Strawberry is propagated from stolons (runners) and transplanted in the form of runner plants. In our field survey conducted from 2019 to 2020, the nested PCR assay detected *C*. *siamense* and *C*. *fructicola* in an average of 20% of symptomless mother plants (Table 2). The percentage of plants latently infected or carrying the pathogen inoculum on surface was > 20% in 6 out of 18 nurseries. This reflects the severe epidemic of strawberry anthracnose in recent years [2,14] and indicates the importance of early detection and removal of latently infected mother plants before they are used for propagation. In 16 out of 18 nurseries, higher detection rates were observed using the culture-based SDEI method, perhaps because the nested PCR assay targets only two of five known pathogenic *Colletotrichum* spp. and only the basal petiole was assayed, whereas the SDEI method nonspecifically detects any viable *Colletotrichum* spp. that forms conidial masses on the whole leaf. In the remaining two nurseries (sites 6 and 10), higher detection rates were observed using the nested PCR assay than the SDEI method. This could be due to more frequent usage of fungicides or the farmers just sprayed fungicides before our sampling. When most *Colletotrichum* spp. were killed, the dead cells could only be detected by the nested PCR assay.

The use of overhead irrigation in strawberry nurseries or open field cultivation often increases the latent *Colletotrichum* spp. infection rate in strawberry runner plants, which leads to disease outbreaks in fruit-producing fields [48,49]. In some strawberry nurseries in Taiwan, the frequency of spraying fungicides can be as high as once every three days during the six-month nursery period. Frequent application of fungicides not only increases production costs but also causes the emergence of fungicide resistance in the pathogen population [22,48]. To prevent the spread of diseases and improve the health of runner plants produced by strawberry nurseries, the Council of Agriculture (COA) in Taiwan has established a voluntary pathogen-free certification system for strawberry propagation in 2018. According to the guidelines, anthracnose is one of the key diseases required to be tested and excluded from strawberry propagation. The nested PCR assay developed in this study has been applied for certification of ‘pathogen-free’ strawberry plants, from which healthy mother plants and runner plants can be supplied to farmers. The use of healthy plant materials combined with integrated control measures will contribute to the production of safe and high-quality strawberries, which is a win-win situation for both producers and consumers.

## Supporting information

S1 Fig Alignment of the spacer region sequences from different *Colletotrichum* spp. The locations of the nested PCR primers in the alignment region (indicated by arrows) are 381–401 bp (Col_nest-1F), 912–930 bp (Col_nest-1R), 510–529 bp (Col_nest-2F), and 645–680 bp (Col_nest-2R). Parts of the sequences of the L-arabinitol 4-dehydrogenase (*ladA*) and NAD(P)H-dependent D-xylose reductase (*xyl1*) genes are also shown.
(TIF)

S1 Table Fungal isolates used in this study. (DOCX)

S2 Table List of *Colletotrichum* spp. genomes used for comparative genomics analysis in this study. (XLSX)

S3 Table List of *Trichoderma* spp. genomes used in this study. (XLSX)

S4 Table List of *Fusarium* spp. genomes used in this study. (XLSX)

S1 Raw images (PDF)
