# Supplementary figures and images for: Development of a nested PCR assay for detecting Colletotrichum siamense and Colletotrichum fructicola on symptomless strawberry plants
Source: PLoS One. 2022 Jun 28;17(6):e0270687. doi: 10.1371/journal.pone.0270687 (PMC9239453; doi:10.1371/journal.pone.0270687)

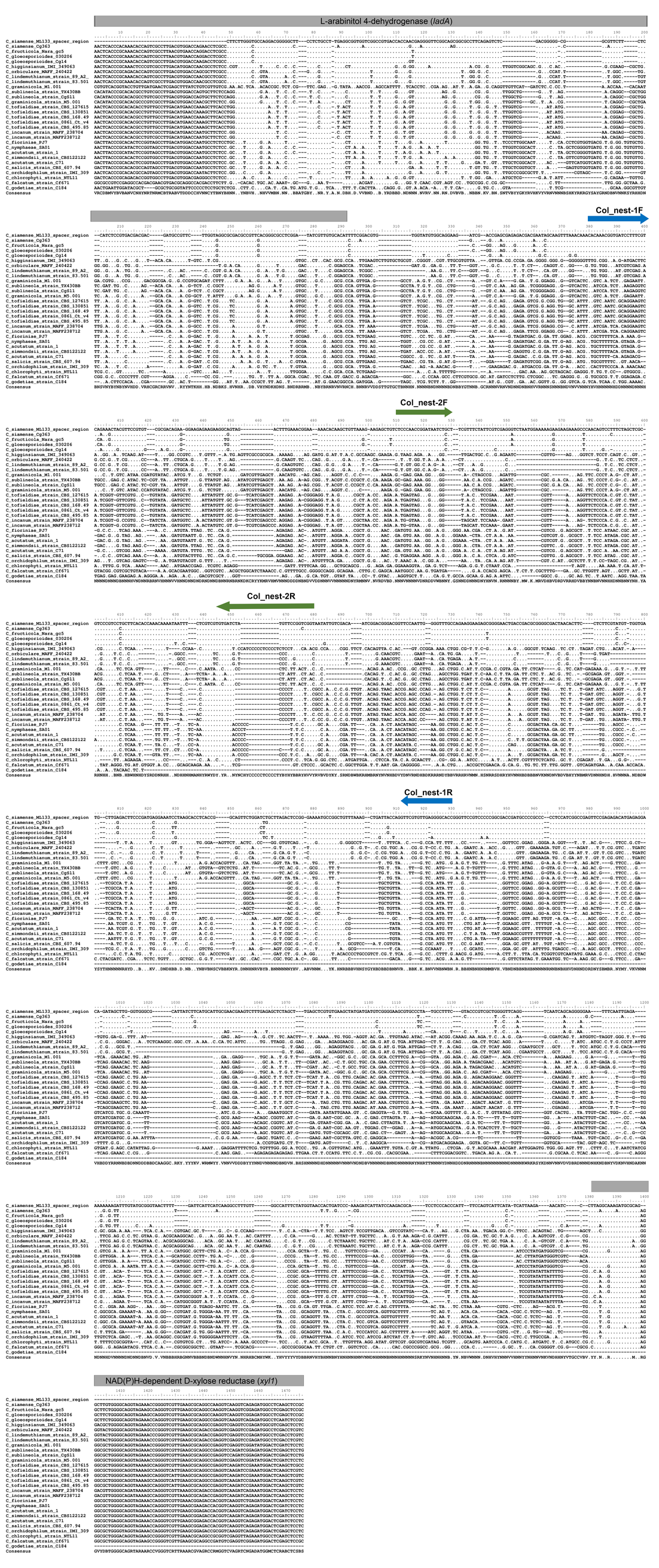

Supplement: S1 Fig — The locations of the nested PCR primers in the alignment region (indicated by arrows) are 381–401 bp (Col_nest-1F), 912–930 bp (Col_nest-1R), 510–529 bp (Col_nest-2F), and 645–680 bp (Col_nest-2R). Parts of the sequences of the L-arabinitol 4-dehydrogenase (ladA) and NAD(P)H-dependent D-xylose reductase (xyl1) genes are also shown. (TIF) [file pone.0270687.s001.tif]

Fig 2

(A)

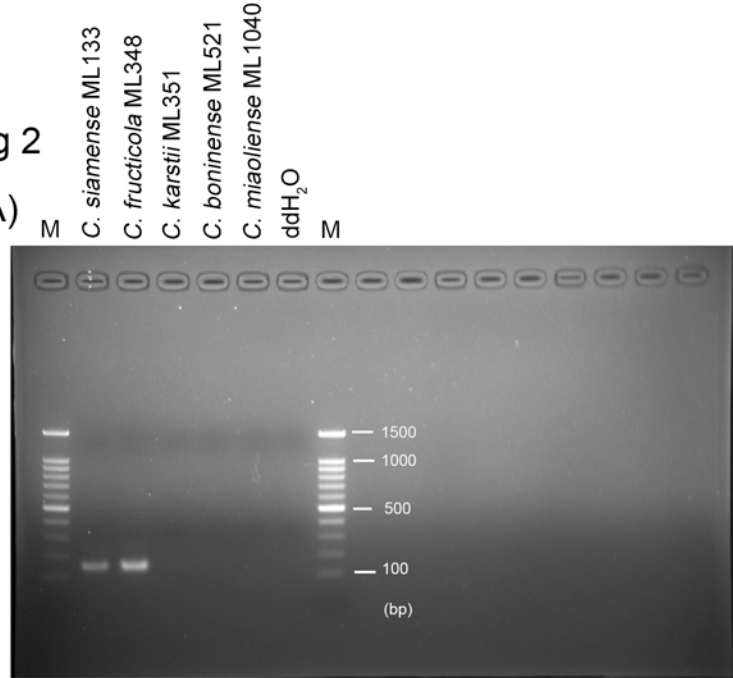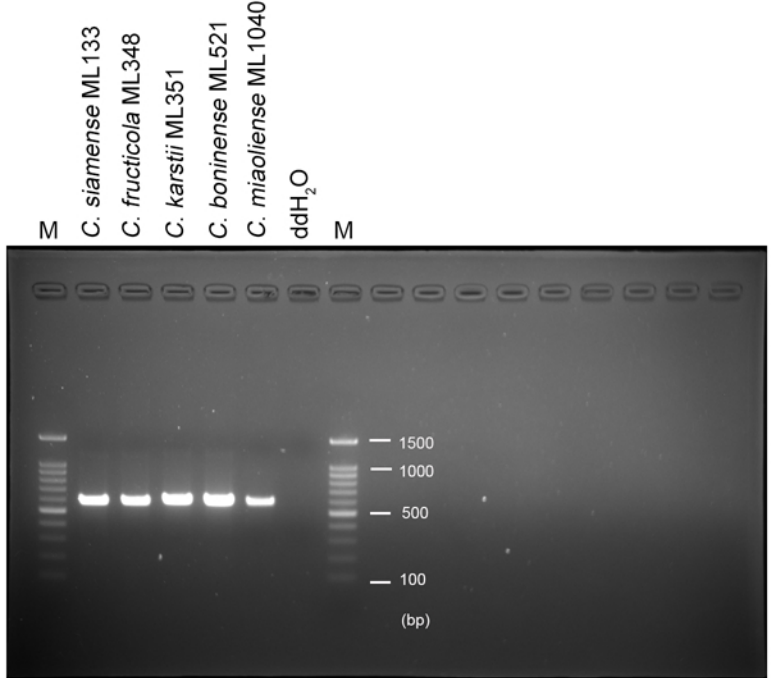

(B)

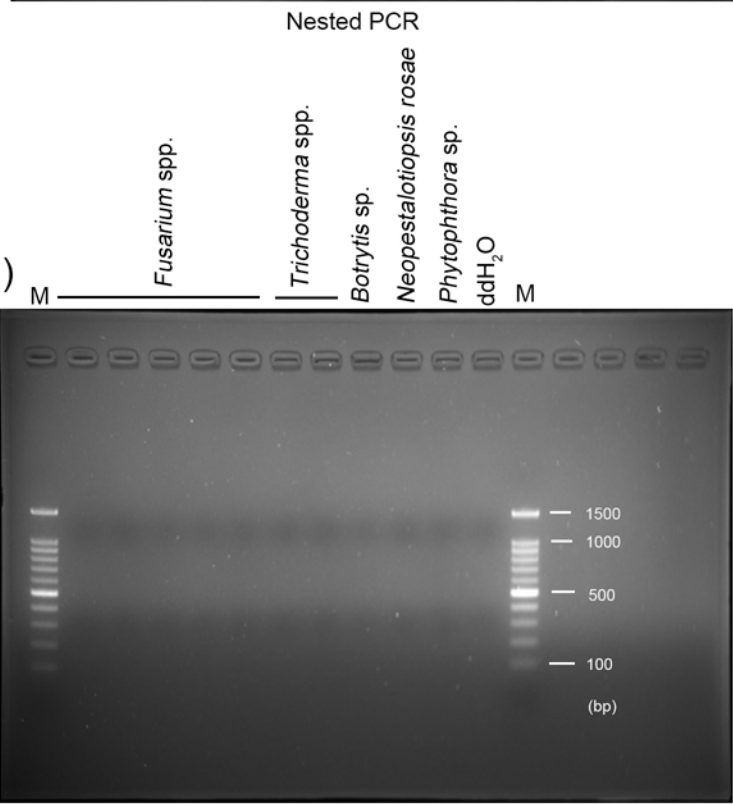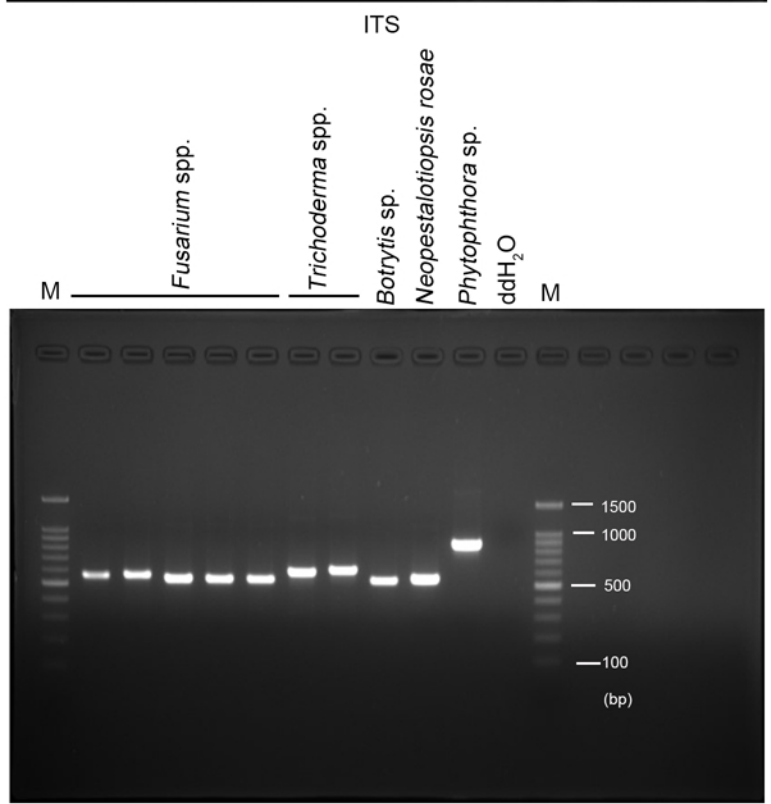

(C)

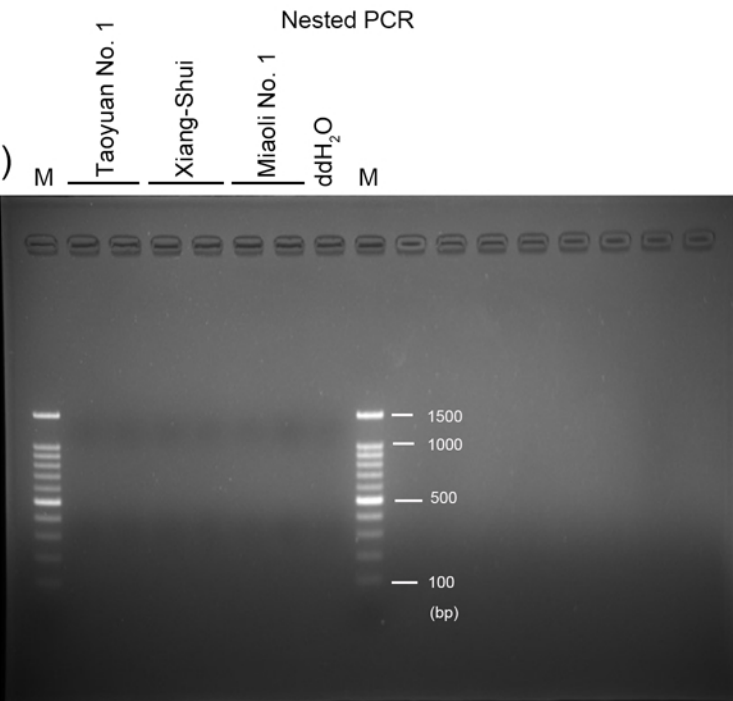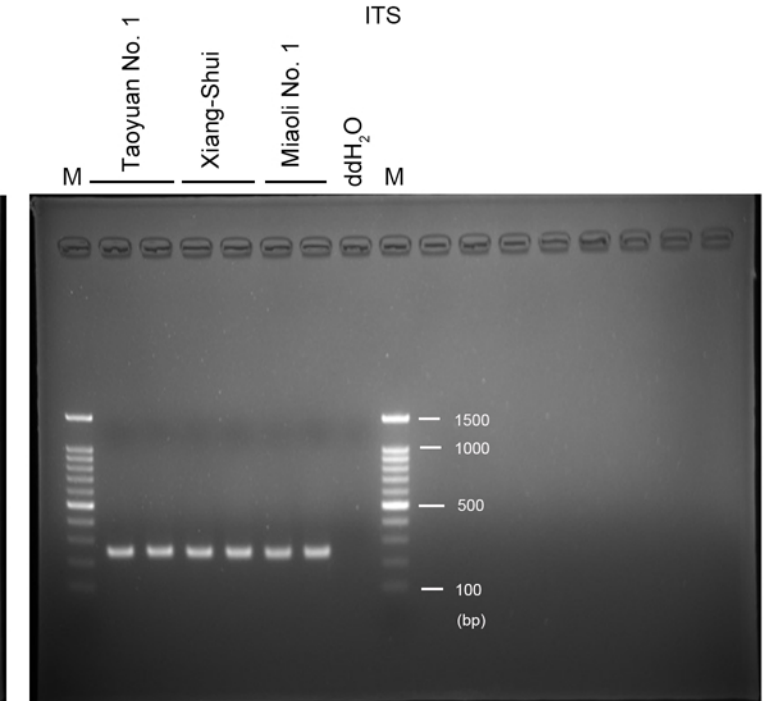

Nested PCR

ACTIN

Fig 3

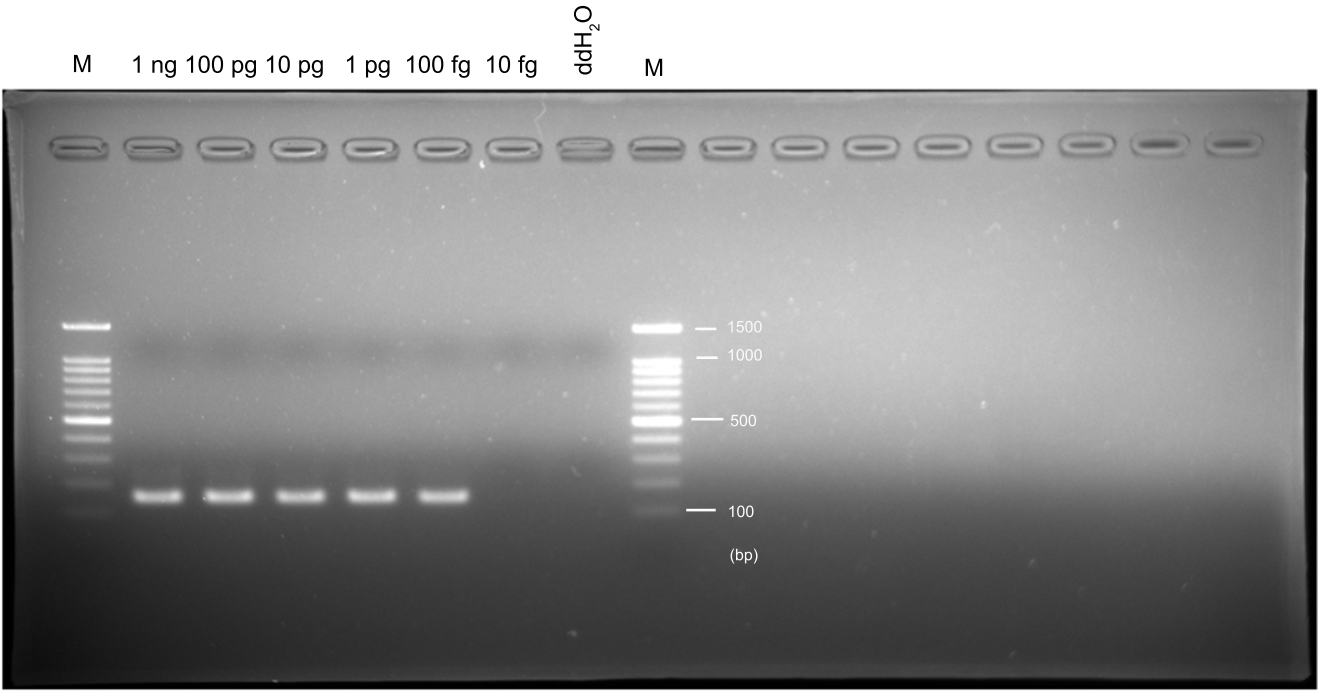

Fig 4

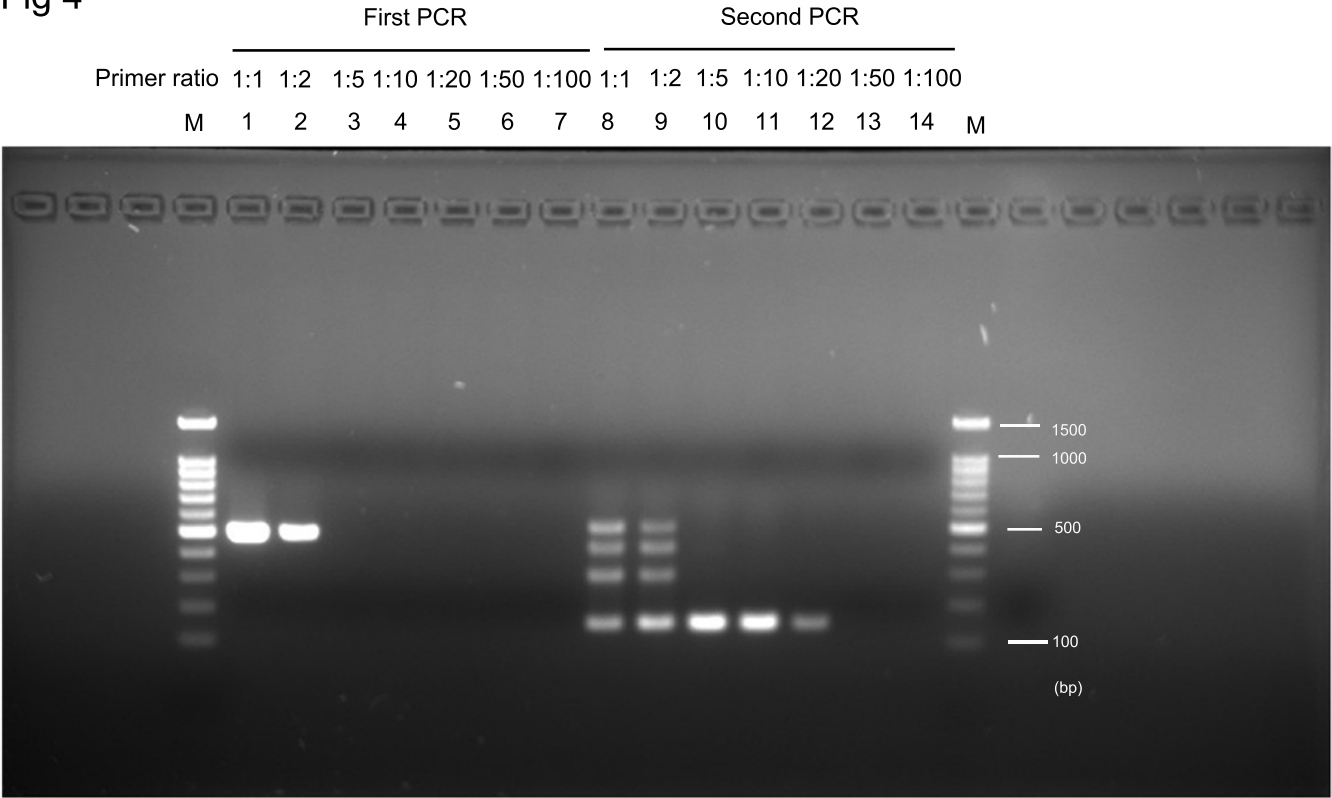

Supplement: S1 Raw images — (PDF) [file pone.0270687.s006.pdf]
