# Supplementary material for: Development of a nested PCR assay for detecting Colletotrichum siamense and Colletotrichum fructicola on symptomless strawberry plants
Source: PLoS One. 2022 Jun 28;17(6):e0270687. doi: 10.1371/journal.pone.0270687 (PMC9239453; doi:10.1371/journal.pone.0270687)
Supplement: S1 Table — (DOCX) [file pone.0270687.s002.docx]

| Species | Isolate | Collection date | Host plant / source | Sampling site | Reference |
| --- | --- | --- | --- | --- | --- |
| *Botrytis* sp. | ML2145 | 2019/02/13 | Strawberry / diseased fruit | Dahu Township, Miaoli County | This study |
| *Colletotrichum boninense* | ML521 | 2013/01/21 | Strawberry / diseased leaf | Taian Township, Miaoli County | [1] |
| *Colletotrichum fructicola* | ML348 | 2012/07/06 | Strawberry / diseased leaf | Shitan Township, Miaoli County | [1] |
| *Colletotrichum karstii* | ML351 | 2012/07/06 | Strawberry / diseased leaf | Shitan Township, Miaoli County | [1] |
| *Colletotrichum miaoliense* | ML1040 | 2016/10/28 | Strawberry / diseased crown | Shitan Township, Miaoli County | [1] |
| *Colletotrichum siamense* | ML133 | 2011/10/28 | Strawberry / diseased crown | Dahu Township, Miaoli County | [1] |
| *Fusarium* sp. | ML292 | 2012/06/27 | Strawberry / diseased root | Taian Township, Miaoli County | This study |
| *Fusarium* sp. | ML432 | 2012/08/22 | Strawberry / diseased crown | Dahu Township, Miaoli County | This study |
| *Fusarium* sp. | ML653 | 2014/07/15 | Strawberry / diseased crown | Dahu Township, Miaoli County | This study |
| *Fusarium* sp. | ML672 | 2015/04/02 | Strawberry / nearby soil (diseased plant) | Dahu Township, Miaoli County | This study |
| *Fusarium* sp. | ML841 | 2016/07/21 | Strawberry / runner (symptomless plant) | Tongluo Township, Miaoli County | This study |
| *Neopestalotiopsis* *rosae* | ML2147 | 2019/02/18 | Strawberry / diseased leaf | Dahu Township, Miaoli County | [2] |
| *Phytophthora* sp. | ML2640 | 2020/09/29 | Strawberry / diseased root | Dahu Township, Miaoli County | This study |
| *Trichoderma asperellum* | ML01 | 2009/05/18 | Grape / rhizosphere soil (symptomless plant) | Zhuolan Town, Miaoli County | [3] |
| *Trichoderma* sp. | ML1425 | 2017/03/15 | Strawberry / petiole (symptomless plant) | Dahu Township, Miaoli County | This study |

**S1 Table. Fungal isolates used in this study**

References

1. Chung PC, Wu HY, Wang YW, Ariyawansa HA, Hu HP, Hung TH, et al. Diversity and pathogenicity of *Colletotrichum* species causing strawberry anthracnose in Taiwan and description of a new species, *Colletotrichum miaoliense* sp. nov. Sci Rep. 2020;10(1). doi: 10.1038/s41598-020-70878-2. PubMed PMID: WOS:000608604500001.

2. Wu HY, Tsai CY, Wu YM, Ariyawansa HA, Chung CL, Chung PC. First report of *Neopestalotiopsis rosae* causing leaf blight and crown rot on strawberry in Taiwan. Plant Dis. 2021;105(2):487-. doi: 10.1094/pdis-05-20-1045-pdn. PubMed PMID: 32976075.

3. Chou H, Xiao YT, Tsai JN, Li TT, Wu HY, Liu LYD, et al. In vitro and in planta evaluation of *Trichoderma asperellum* TA as a biocontrol agent against *Phellinus noxius*, the cause of brown root rot disease of trees. Plant Dis. 2019;103(11):2733-41. doi: 10.1094/pdis-01-19-0179-re. PubMed PMID: 31483183.
